# Dual chemotaxis strategy of bacteria in porous media: Run-time and turn-angle bias

**DOI:** 10.1016/j.bpj.2026.06.002

**Published:** 2026-06-04

**Authors:** Sönke Beier, Veronika Pfeifer, Agniva Datta, Robert Großmann, Carsten Beta

**Affiliations:** 1 Institute of Physics and Astronomy, University of Potsdam, Potsdam, Germany; 2 Nano Life Science Institute (WPI-NanoLSI), Kanazawa University, Kanazawa, Japan

## Abstract

Chemotaxis of bacterial swimmers that move in a run-and-turn pattern is well studied in uniform bulk fluid. It is primarily based on modulating the run time in dependence on the swimming direction with respect to the source of chemoattractant (run-time bias). Here, we provide evidence that the lophotrichously flagellated soil bacterium *Pseudomonas putida* may also perform chemotaxis in porous media where the free path length is severely restricted. Besides the classical run-time bias, we identify a second chemotaxis strategy, the turn-angle bias: the change in swimming direction upon a turn event is adjusted so that the orientation of the subsequent run phase is more likely to be directed toward the source of chemoattractant. We show that the resulting directional preference of runs arises from active, motor-induced turning events. Passive mechanical trapping in the porous matrix, in contrast, weakens the resulting preference of run orientations. Agent-based simulations, which rely on experimentally observed statistical properties of swimming patterns, indicate that the turn-angle bias is the predominant chemotaxis strategy of bacteria in porous environments.

## Significance

Chemotaxis is the ability to navigate toward or away from chemical sources, seeking nutrients or avoiding harmful substances, essential for survival and growth. To follow a chemical gradient, bacteria with run-and-turn motility are commonly assumed to adjust their run times depending on how their swimming direction is oriented with respect to the gradient. In this work, we broaden the traditional picture of bacterial chemotaxis, providing the first direct experimental evidence that bacteria in disordered media also bias their turn angles to navigate toward a nutrient source. By combining experiments and model simulations, we reveal that the turn-angle bias becomes the dominant chemotaxis strategy under tight spatial constraints, leading to an improved understanding of bacterial chemotaxis in real-world environments.

## Introduction

Bacteria sense their environmental conditions and may respond to chemical stimuli by chemotaxis, the directed movement toward or away from a chemical source.^1^ Despite its high metabolic and energetic cost, about half of the known bacterial species can perform chemotactic motion.^2^^,^^3^ It provides significant benefits as the directed motion along a chemical gradient allows bacteria to access nutrients and to guide culture expansion toward more favorable conditions.^4^^,^^5^^,^^6^ Typically, bacteria swim in straight runs interrupted by short reorientation events. To bias their direction of motion, bacteria modulate the frequency of reorientation events, leading to longer runs when swimming toward a chemoattractant source.^7^ This is known as the run-time bias. In complex and confined habitats such as tissues and sediments, swimming motility and chemotaxis are strongly affected by the geometry of the environment.^8^^,^^9^ Nevertheless, in these surroundings, directed movement is also a key prerequisite for many essential processes such as tissue infection^10^ or symbiosis with plants in the rhizosphere.^11^ In confined environments, however, runs are restricted by the free path length, so that bacteria frequently collide with the surrounding matrix or become trapped.^12^^,^^13^^,^^14^^,^^15^^,^^16^ Here, flagellar activity that reorients the cell body is crucial for finding the exit of a cavity and escaping from traps,^17^^,^^18^^,^^19^ while physiological adaptations in flagellar architecture may further help to move within a porous environment.^20^ If the mean free path length matches the average run length, bacteria reorient frequently enough to escape from traps, but runs are, at the same time, long enough to enable efficient spatial exploration.^14^^,^^17^^,^^21^^,^^22^ In very dense environments, in contrast, run lengths are severely reduced; in consequence, a chemotaxis strategy that is based on modulating the run length becomes inefficient.^9^

An alternative mechanism for achieving chemotactic drift is the turn-angle bias: cells actively modulate the changes in swimming direction induced by turn events, depending on the gradient direction. To navigate toward a source of chemoattractant, cells swimming away from the source will, on average, perform larger turns than cells swimming toward it. For *Escherichia coli* (*E. coli*) in uniform liquid, the angular bias was first theoretically predicted^23^ and later experimentally demonstrated.^24^ Subsequent studies confirmed the angle bias for *E. coli*^25^^,^^26^ as well as for the peritrichously flagellated bacterium *Salmonella* Typhimurium under bulk liquid conditions.^27^ It was also proposed that the turn-angle bias may contribute to *E. coli* chemotaxis in porous media, where an orientational preference in the directions of bacterial runs was observed.^9^

To elucidate the relevance of a turn-angle bias to chemotaxis in disordered confined surroundings, we focus on the soil bacterium *Pseudomonas putida* (*P. putida*). The natural habitat of *P. putida* is a crowded, densely packed granular soil environment, where *P. putida* colonizes plant roots and shows chemotaxis toward root exudates.^28^ *P. putida* thus appears as a natural candidate to address the question of chemotactic navigation under confinement.^29^^,^^30^ In aqueous bulk solution, the lophotrichously flagellated *P. putida* cells exhibit a complex multi-mode swimming pattern composed of three different run types: the flagellar bundle may push, pull, or wrap around the cell body.^31^^,^^32^ Run phases are interrupted by stops and reversals in the swimming direction, resulting in small and large turning angles, respectively.^31^ In the presence of a chemoattractant gradient, the swimming pattern in uniform liquid surroundings exhibits a directional drift induced by a run-time bias during wrapped mode swimming.^33^

To address the question of chemotaxis strategies in porous media, we focus on *P. putida* chemotaxis in disordered matrices with varying concentrations of agar. In a classical swimming agar assay, bacteria are injected into semisolid agar and spread radially around the injection point within several hours. This can be described as a spreading process based on random swimming motility, cell growth, and division.^17^ Chemotaxis can additionally promote the spreading of a culture.^34^

For *P. putida*, it was shown that the swimming pattern in agar consists of straight-run phases interrupted by events during which the bacterium stops and reorients.^13^^,^^14^ These events can be divided into two categories. On one hand, there are motor-induced, actively triggered short stops and directional reversals, known from the motility in bulk fluid, which we will refer to as turn events with average turning angles of 0° and 180°, respectively.^31^ On the other hand, there are longer-lasting trap events, which were observed in agar and originate from mechanical arrest in the porous network.^13^^,^^14^

In this work, we characterize the chemotaxis strategy of *P. putida* in an agar network. We provide direct evidence that *P. putida* cells actively adjust their turning angles depending on their current swimming direction to navigate chemical gradients in confined environments. We further show that chemotactic navigation of *P. putida* in the porous agar matrix predominantly relies on this turn-angle bias, which is induced by active turn events.

## Materials and methods

### Cell culture

For the experiments, the strain *P. putida* KT2440_S267C_ FliC was used.^32^ *P. putida* was grown overnight in a shaking culture at 30°C in LB medium.

### Swimming agar assay with externally imposed gradient

To visualize chemotaxis macroscopically, a chemotaxis agar assay was used.^35^ For this purpose, 30 mL M8 (6 g/LNa_2_HPO_4_, 3 g/LKH_2_PO_4_, and 0.5 g/L NaCl) agar plates with 0.2% glucose and 1 mM MgSO_4_ as final concentration were poured into standard Petri dishes (*∅* 85 mm, height 15 mm). Plates solidified over 4 h. 10 μL casamino acids were injected into the agar. The plates were kept overnight to create the chemoattractant gradient. After around 16 h, 2 μL of cells with an OD_600_ of around 1 were injected into the agar at distances ranging from 0.5 to 3.5 cm away from the casamino acids injection point. The effect of the nonisotropic spreading could be observed after 1–2 days. The effect could be seen for all tested distances between the chemoattractant and the culture injection point.

### Swimming agar assay with self-generated gradient

We used the plate-based assay described in Ha et al.^36^ M8 agar plates containing 0.2% glucose, 0.5% casamino acids, and 1 mMMgSO_4_ as final concentration were poured into Petri dishes. Plates solidified over 4 h. 2 μL of cells with an OD_600_ of around 1 were injected into the agar. The agar plates were incubated overnight at room temperature. The trajectories of bacteria were recorded at the edge of the spreading colony around 20 h after injection of the bacteria. Due to the consumption of casamino acids, the bacteria establish a concentration gradient extending from the inside of the colony toward the surrounding medium. Given the short imaging times, it can be assumed that the gradient remains temporally stable during individual recordings. While the concentration gradient will not be linear in our assay, it serves as a realistic approximation of concentration profiles that may be expected in naturally occurring porous environments.

### Microscopy

An inverted microscope (Olympus IX71) was used for the microscopy recordings. An ORCA-Fusion BT Digital CMOS camera from Hamamatsu was used for recordings together with the Hokawo software (Hamamatsu). Phase-contrast images were recorded 30 μm above the bottom surface with a 20× UPLFLN-PH objective (Olympus) at a frame rate of 20 frames per second. The individual recordings had a total length of 1 min.

### Cell tracking

Segmentation and cell tracking were performed using custom-made computer code described in Theves et al.,^31^ which is based on the algorithm presented in Crocker and Grier.^37^ Short trajectories (duration less than 1.5 s) and trajectories with small total displacement (less than 1 μm) were filtered out. For each agar concentration, 10 1-min recordings from different plates and from 2 different days were used and analyzed. First, each recording was processed individually to confirm that fluctuations in the local cell density did not influence the outcome of our analysis. After confirming that analyses of the different replica experiments yielded consistent results, the data from the 10 recordings were pooled into a single large data set for each agar concentration. We recorded a total of 17,703 trajectories for 0.25% agar with a median duration of 66 frames and 18,714 trajectories for 0.3% agar with a median duration of 79 frames.

### Event detection in agar

We used the method described in Datta et al.^14^ to differentiate run phases from stop, turn, and trap events: *k*-means clustering, in combination with a minimum distance of 2 μm between different events, was used to distinguish runs and events. For clustering, the change in direction of motion at a given data point over a shorter time interval (three frames), the change in direction of motion over a longer time interval (five frames) at that data point, and the speed of motion approaching and departing that data point were used. Trajectories were not smoothed.

### Measurements and event detection in bulk fluid

The data and the event detection presented in Alirezaeizanjani et al.^33^ were used to analyze chemotaxis in bulk liquid. Information on the methodology can be found there.

### Agent-based simulation

For agent-based simulation, 5,000 bacteria were simulated over a period of 30,000 time steps, equivalent to 25 min in the biological experiment. The experimentally measured run-time distribution, turn-angle distribution, event-duration distribution, and mean run speed were used to model motility patterns of bacteria. The modeling framework was discussed in Datta et al.^38^; the data and inference techniques were also examined and are shown in Datta et al.^14^ We extended the modeling framework proposed in Datta et al.^38^ to simulate chemotaxis as follows. For the simulations with angular bias, the turn-angle distribution was divided into angular changes after upgradient and downgradient runs; the corresponding random numbers were drawn from these distributions accordingly. For the run-time bias, data were treated analogously: runs were divided into subgroups (upgradient versus downgradient runs) and run-time distributions were inferred and used separately. Further simulation details are discussed in the supplemental information.

## Results

### *P. putida* performs chemotaxis in semisolid agar

**Figure 1:**
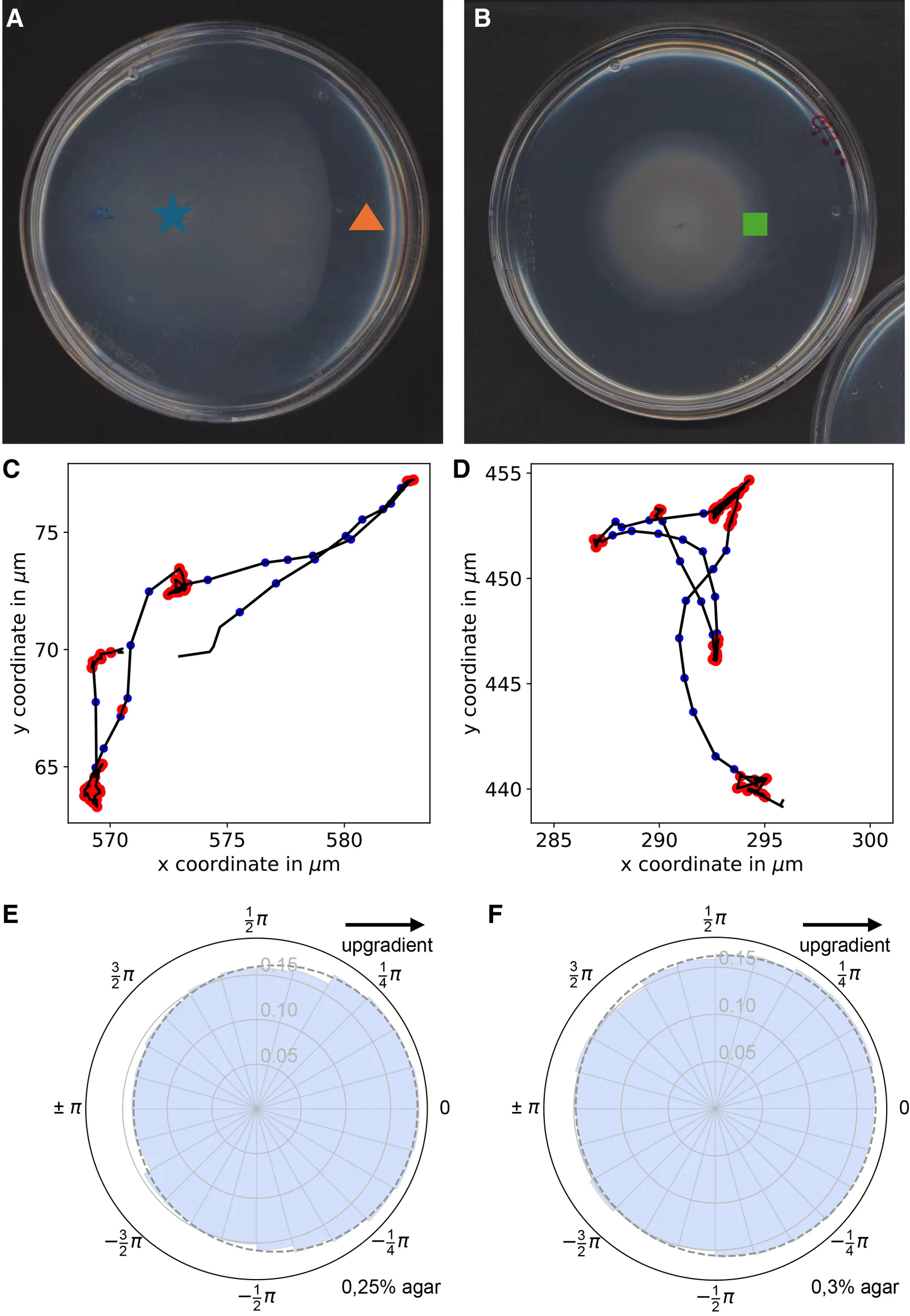
Chemotaxis of *P. putida* in agar at the colony and trajectory level. Macroscopic spreading of a cell colony in 0.3% agar with (A) an externally induced chemoattractant gradient and (B) an initially evenly distributed chemoattractant. In (A), the spreading is anisotropic because bacteria move directionally toward the source of chemoattractant (injection point: orange triangle). Diameter of the plates: 85 mm. In (B), bacteria consume the chemoattractant, resulting in a self-generated gradient. Images in the middle row show exemplary bacterial trajectories in (C) 0.25% and (D) 0.3% agar, recorded at the edge of the expanding colony (green rectangle in B). Run phases are marked in blue and turn and trap events in red. (E) and (F) display probability densities of directions *ϕ* of all displacements of bacteria during run phases in (E) 0.25% and (F) 0.3% agar, respectively. Chemotaxis of bacteria is reflected at the trajectory level by biased motion toward the right (E: 54.2% ± 0.02% and F: 52.4% ± 0.02%). Error bars, obtained by bootstrapping,^39^ represent 2*σ* intervals. Dashed lines represent fits to the data obtained via Fourier expansion to first order, *p*(*ϕ*) ≈ [1 + 2|*f*_1_| cos(*ϕ* − *ψ*)]/(2*π*), where the first nontrivial mode *f*_1_ is another measure for the chemotactic bias (E: |*f*_1_| = 0.067 and F: |*f*_1_| = 0.036).

We first performed a macroscopic spreading experiment to examine whether *P. putida* performs chemotaxis in semisolid agar. For this purpose, we injected casamino acids into the agar to create a gradient via diffusion. Cells were afterward injected some distance away from the chemoattractant source (see materials and methods for details). Figure 1A shows that asymmetric spreading of the colony in the direction toward the source of chemoattractant was observed. This indicates that *P. putida* performs chemotaxis in the porous agar gel. Note that the directional bias toward the attractant in this setting may be partly due to cell growth. At the back of the colony, less casamino acids were present, leading to a decreased growth rate, which may affect the culture’s spread.^17^ Thus, the preferred spreading direction may not only depend on motility but also on local differences in growth rate.^34^ However, a chemotactic drift was also observed at the level of single-cell trajectories. As cell division does not play any role on the timescale at which individual cell trajectories were recorded, we conclude that a chemotactic drift exists in our data.

When chemoattractants were evenly distributed in the agar network, the bacterial colony spreads isotropically (see Figure 1B). The expansion rate depends on the size of the pores as well as the motility and growth rate of bacteria.^13^^,^^17^^,^^18^^,^^34^ In addition, a gradient of chemoattractant was created at the edge of the colony, as the casamino acids were consumed by the bacteria.^17^

Furthermore, we recorded single-cell trajectories of *P. putida* in agar and subdivided them into runs and reorientation events based on the approach described in Datta et al.^14^ Exemplary trajectories are shown in Figures 1C and 1D. We analyzed microscopy data from the edge of a spreading colony (green rectangle in Figure 1B), where bacteria create their own chemoattractant gradient from the inside to the outside of the colony by local consumption of casamino acids.

The angular histograms in Figures 1E and 1F show that bacteria were more likely to swim upgradient than downgradient during run phases. Thus, at the level of single-cell trajectories, we also confirmed that *P. putida* performed chemotaxis in both tested agar concentrations. For the lower agar concentration of 0.25%, the bias was more pronounced than for 0.3%. Similar results were observed for the setup shown in Figure 1A.

### A run-time bias is observed in agar, despite the limited mean free path length

The observation of chemotaxis, both at the colony and trajectory levels, raises the question which chemotaxis strategy is employed by *P. putida* to achieve directed motion in agar. In the agar gel, *P. putida* faces a disordered environment with an average pore size in the range of 740–4,800 nm for 0.25% agar.^18^ This is comparable to the observed mean run length *ℓ* ≈ 8 μm of the bacterial swimmers in the gel.^14^ As the mean run length and time in bulk liquid are one order of magnitude larger,^33^ we conclude that runs in the agar gel are severely restricted due to spatial confinement (see Table 1).

**Table 1:** Mean run times for upgradient and downgradient runs as well as all runs combined

	*τ*_all_	*τ*_up_	*τ*_down_
0.25% agar	0.299 s	0.304 s	0.293 s
0.3% agar	0.232 s	0.234 s	0.229 s

The statistical uncertainty was estimated via bootstrapping^39^ to be 0.003 s, corresponding to a 2*σ* confidence interval.

**Figure 2:**
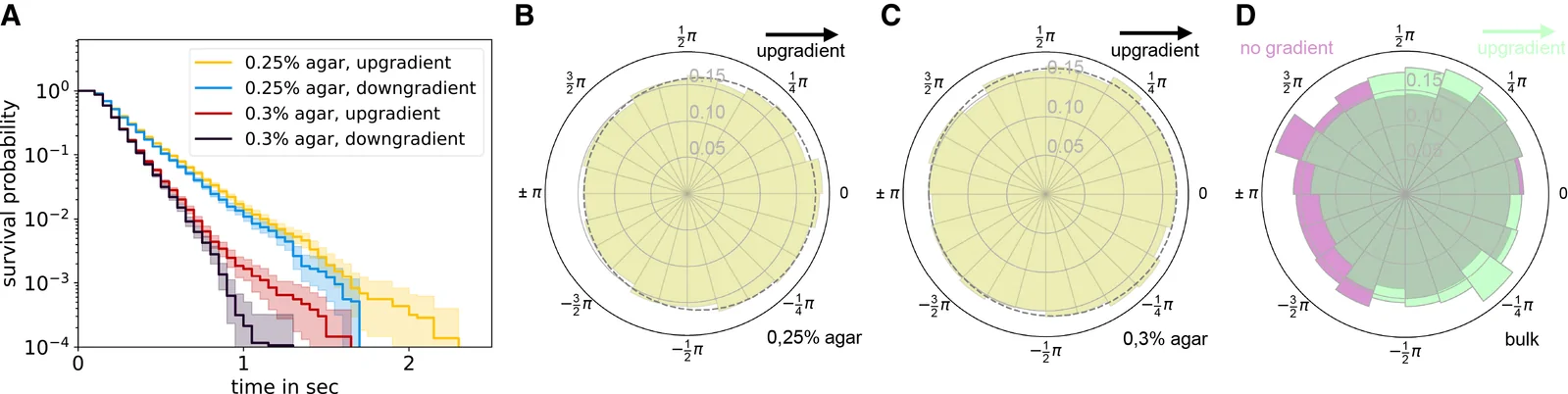
Chemotaxis strategy of *P. putida*: run-time bias and orientational preference of runs. (A) Run-time distributions for different agar concentrations, represented as survival probabilities, i.e., the probability that a run is longer than a given time, obtained by nonparametric maximum likelihood estimation.^40^ Error bars show a 2*σ* interval, calculated by bootstrapping.^39^ (B) and (C) represent the probability densities of the orientations of runs (orientation of the displacement vector from the first to the last data point of each run). (B) 53.3% ± 0.3% of 68,431 runs (from 17,703 tracks) in 0.25% agar are oriented upgradient (to the right in this case). The dashed line represents a fit to the data obtained via Fourier transform to first order, *p*(*ϕ*) ≈ [1 + 2|*f*_1_| cos(*ϕ* − *ψ*)]/(2*π*), where the first nontrivial mode |*f*_1_| = 0.054 is a measure of the strength of the bias. In 0.3% agar (C), 51.9% ± 0.3% of 73,531 runs (from 18,714 tracks) are oriented upgradient; corresponding fit parameter: |*f*_1_| = 0.029 (dashed line). (D) Probability densities of the orientations of runs, recorded in bulk fluid (data taken from Alirezaeizanjani et al.^33^). Light green histogram: orientation of runs in the presence of a gradient (2,450 runs and 1,378 tracks); 54.8% ± 1.1% of runs are pointing in the upgradient direction. The dark magenta histogram shows the control experiment without a gradient (3,363 runs and 1,799 tracks); no significant bias is observed in the absence of the gradient in a control experiment (49.4% ± 1.0% of runs are oriented upgradient).

To test whether the commonly observed run-time bias also plays a role in chemotaxis in agar gels despite the limited mean free path length, we analyzed the durations of runs pointing upgradient and downgradient separately. In Figure 2A, the corresponding survival probabilities, i.e., the probability that a run is longer than a given time, are shown. They approximately exhibit an exponential decay. The mean run time strongly depends on the agar concentration: a shorter mean free path length in the denser agar network results in a significantly shorter mean run time in 0.3% agar than in 0.25% agar (see Table 1). Even though runs are tightly restricted in the agar gel, comparing run-time statistics for up- and downgradient runs reveals a small run-time bias for both agar concentrations; runs upgradient are indeed longer than downgradient runs on average.

Based on the mean run times of upgradient runs *τ*_up_, downgradient runs *τ*_down_, and all runs in one agar concentration *τ*_all_, as shown in Table 1, the strength of the run-time bias can be quantified as $S=\left(\tau_{\text{up}}-\tau_{\text{down}}\right)/\tau_{\text{all}}$. We find *S*_0.25_ = 0.037 ± 0.004 for 0.25% agar and *S*_0.3_ = 0.022 ± 0.002 for 0.3% agar; accordingly, the strength of the run-time bias decreases with increasing agar density. Notably, the average time is much shorter than the mean run time in bulk fluid.^31^^,^^33^

### Runs are more likely to point toward increasing chemoattractant concentration both in agar and bulk liquid

Despite the restricted mean free path length in the porous agar matrix, a run-time bias was detected. Is this the only chemotaxis strategy at work, or is *P. putida* relying on additional mechanisms to enhance the directional bias? We test for a run-orientation preference by computing histograms showing the number of runs depending on their direction with respect to the chemoattractant gradient (see Figures 2B and 2C). The histograms show that, in addition to the run-time bias, runs are more likely to point in the upgradient direction than against it. This suggests that *P. putida* may actively induce a turn-angle bias, increasing the probability that a run is directed toward increasing chemoattractant concentration after a turn event. The anisotropy in the histogram was observed for both agar concentrations, but it is more pronounced in the less dense agar network. Specifically, 53.3% ± 0.3% of runs were directed in the upgradient direction for 0.25% agar, while this was only the case for 51.9% ± 0.3% of runs for 0.3% agar. Note that Figures 1E and 1F show the orientation of all individual displacements during runs from one frame to the next, whereas Figures 2B and 2C represent the histograms of the mean run orientations, i.e., the number of runs in a certain direction. Therefore, Figure 1 only indicates whether chemotaxis is present, not the underlying chemotaxis strategy—the bias may be due to run-time or turn-angle bias. In contrast, Figures 2B and 2C enable us to conclude that there are more upgradient than downgradient runs, which indicates the presence of a turn-angle bias.

We tested whether a similar bias is also observed for chemotaxis in bulk fluid. For this purpose, we reanalyzed the data from Alirezaeizanjani et al.^33^ and found a similar anisotropy in the histogram of run directions (see Figure 2D). For the bulk fluid data, the results are less clear, as the total number of observed runs is much lower. Nevertheless, there is a clear difference in the number of runs pointing upgradient between the experiment with a nutrient gradient (54.8% ± 1.1%) and the control experiment without a gradient (49.4% ± 1.0%). We may thus conclude that the bias of run directions does not exclusively arise in a porous environment but is an intrinsic element of the chemotaxis machinery of *P. putida*.

### A turn-angle bias induces the orientational preference of runs in chemoattractant gradients

**Figure 3:**
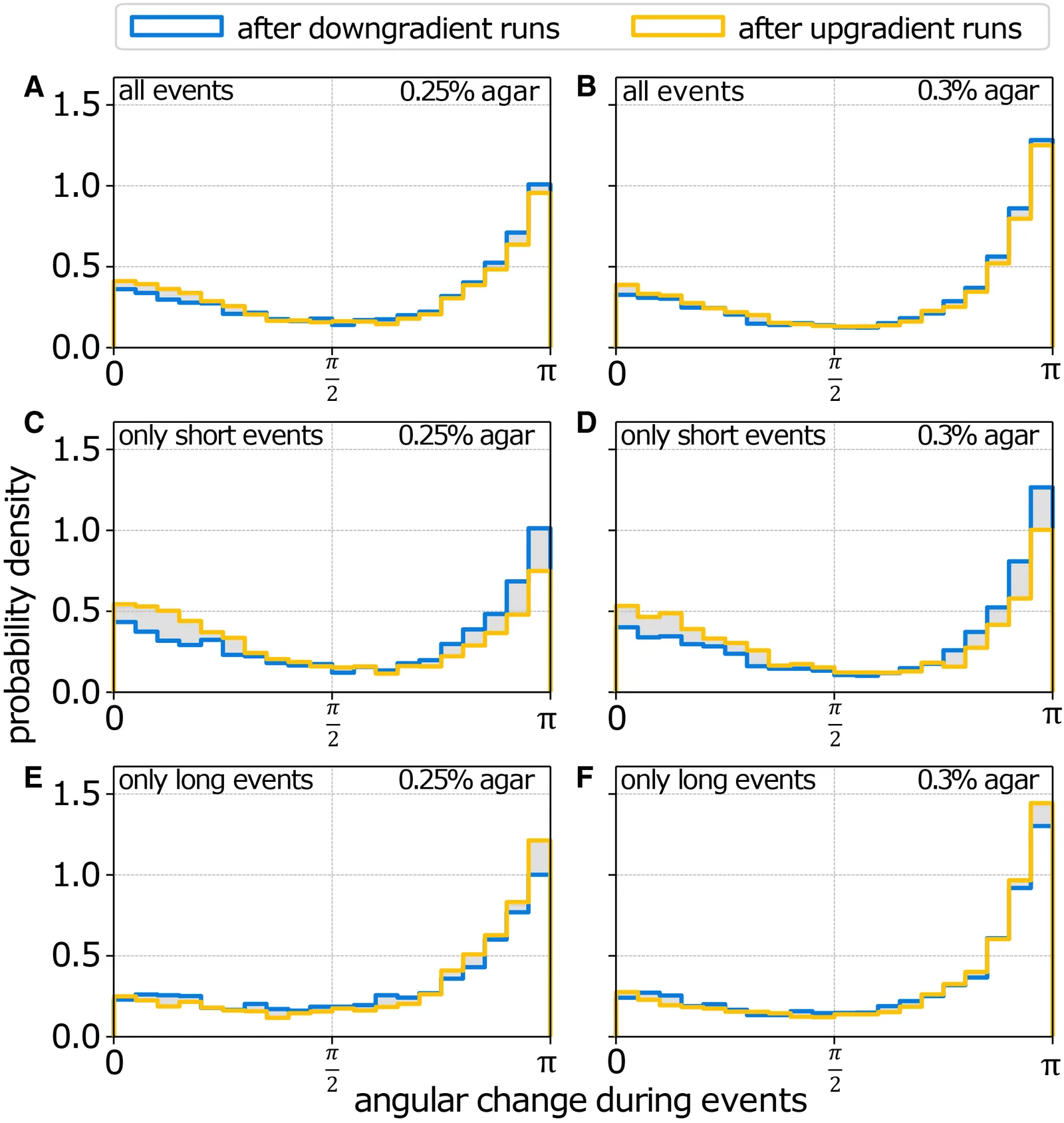
Direct evidence for a turn-angle bias during *P. putida* chemotaxis resulting from short, motor-induced turn events. Histograms of angular change during reorientation events in dependence on the direction of the preceding run in 0.25% agar (left column) and in 0.3% agar (right column), respectively. (A) and (B) include all observed events—only a small difference between turn angles following upgradient (yellow) and downgradient (blue) runs appears in 0.25% agar. We further subdivided events according to their duration into short (duration ≤1s; C and D) and long (duration >1s; E and F) events. While there is no turn-angle bias for long events, the angular reorientation during a short event significantly depends on the direction of the preceding run. Therefore, we conclude that short events, which are more likely to be actively triggered, motor-induced turns, contribute a turn-angle bias to the overall chemotaxis strategy of *P. putida*, whereas long events are dominated by spatial constrictions in the disordered agar gel and thus show no directional dependence.

In order to achieve a directional preference, i.e., a higher fraction of runs pointing toward the source of chemoattractant, cells have to actively tune their directional reorientation during a turn event depending on the direction of the preceding run phase (turn-angle bias). Indeed, our data indicate that if a bacterium swims toward increasing nutrient concentration (upgradient), the probability of observing small turning angles during the subsequent turn event is slightly increased, thus elevating the probability that the bacterium continues to swim toward the nutrient source. On the other hand, runs in the downgradient direction were more often followed by turn events with a large change in direction of motion, increasing the probability that the bacterium would turn toward the nutrient source (see Figures 3A and 3B for 0.25% and 0.3% agar, respectively). Even though the differences between the histograms of turn angles following upgradient and downgradient runs are small in 0.25% and negligible in 0.3% agar, we may conjecture that *P. putida* actively modulates its turning angles, at least for some of the turn events.

### The turn-angle bias results from motor-induced turn events, not from passive mechanical trapping

It is known from previous studies of swimming motility in open bulk liquid that the runs of *P. putida* are interrupted by stops and directional reversals, i.e., turn events with average turning angles of 0° and 180°, respectively.^31^ The duration of these actively triggered, motor-induced turning maneuvers is 0.3 s on average.^33^ In the agar matrix, however, passive mechanical trapping is observed in addition, resulting in an average turn event duration of approximately 2–4 s, depending on the agar concentration.^14^

To individually analyze the impact of motor-induced turning maneuvers and mechanical trapping on the angle bias, we separated the reorientation events according to their durations into short (duration <1s) and long (duration >1s) turn events and considered their turn-angle distributions separately (Figure 3).

**Figure 4:**
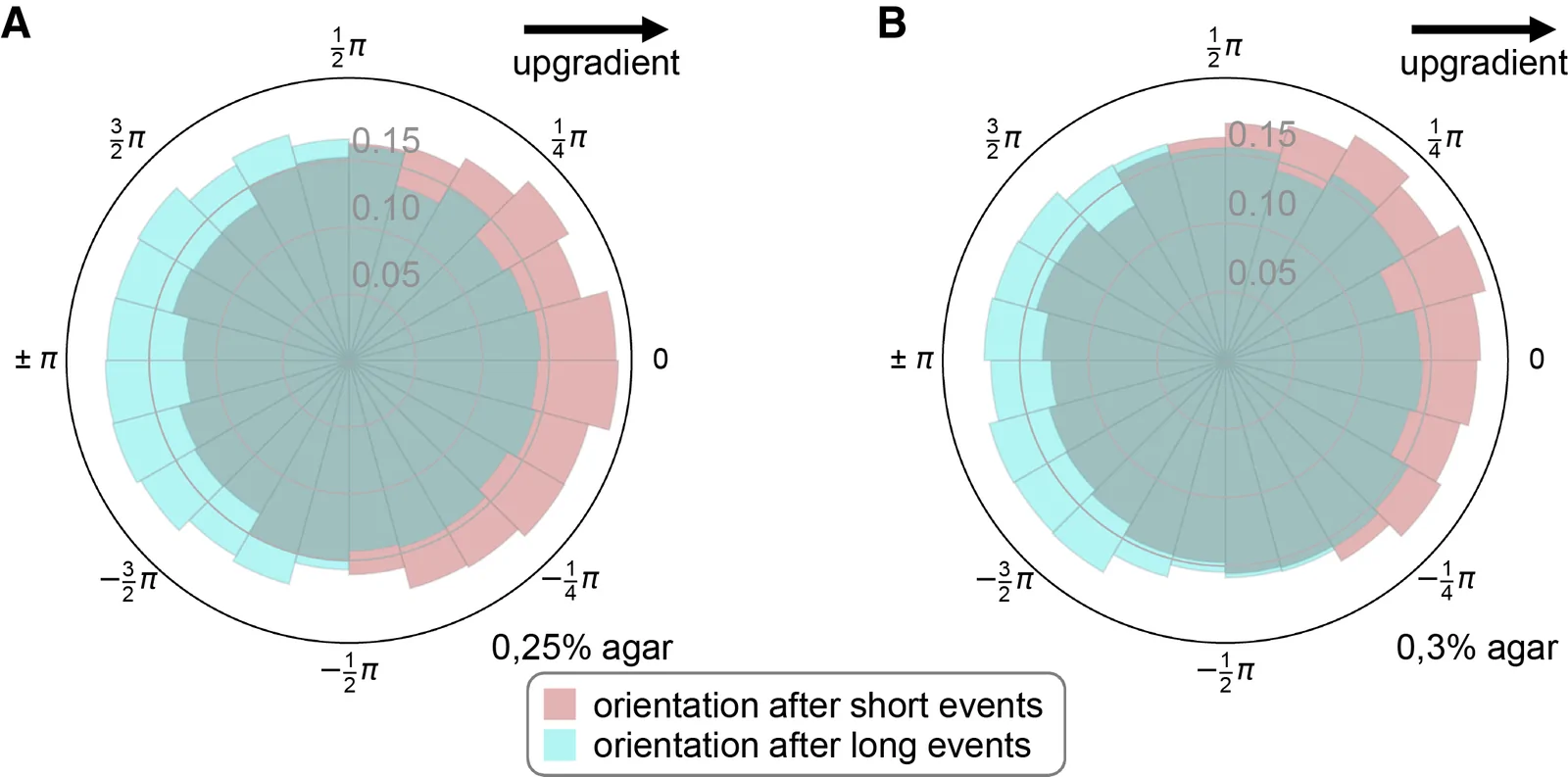
Run orientation in dependence on preceding event duration. The histograms show the probability density of run orientations, where runs are split into two subgroups: runs following a short event (duration ≤1s) in dark red and runs following a long event (duration >1s) in light blue. (A) 0.25% agar and (B) 0.3% agar. Fraction of runs, oriented upgradient, following a short event: (A) 56.7% ± 0.4% of 38,376 runs and (B) 55.6% ± 0.4% of 36,351 runs. Equivalent fractions of runs following a long event: (A) 44.9% ± 0.5% of 17,582 runs and (B) 46.8% ± 0.4% of 25,613 runs.

We found that the turn-angle bias can be attributed exclusively to the short turn events in both agar concentrations (see Figures 3C and 3D), while the turn-angle distribution does not depend on the direction of the preceding run for long events, as shown in Figures 3E and 3F. This is supported by the results displayed in Figure 4: runs after short turn events are more likely to point toward the nutrient source. We thus conclude that actively triggered, motor-induced turns are required to induce a turn-angle bias.

Note that, unlike runs following short events, runs after long mechanical trapping events in the matrix are more likely to point away from the chemoattractant source (Figure 4). This can be explained by the typical trap geometry. If a bacterium gets trapped, it is located in a narrow cavity that, in many cases, has only a single exit through which the cell has entered the trap. As a result, cells often change their run orientation strongly after a trap event because they have to reverse their swimming direction to leave the trap.^14^ As overall more runs are pointing in gradient direction, the large angular change after a trap event thus induces a tendency that the following run points away from the chemoattractant source.

### Numerical simulations reveal importance of turn-angle bias

In order to assess the importance of the two chemotaxis mechanisms—turn-angle bias and run-time bias—for the overall chemotactic drift, we performed agent-based simulations of chemotactic spreading of a bacterial population. Simulations are based on our recently proposed model of intermittent active motion,^38^ which we successfully applied to describe the diffusion of bacteria in a porous gel.^14^ Here, we extend this modeling framework to simulate the spreading of an ensemble of bacteria in a chemoattractant gradient. Specifically, we compare bacteria with both run-time and turn-angle bias to bacteria with turn-angle bias only, with run-time bias only, and, as a control, without any chemotactic response. Model parameters are estimated from experimental data, such as the experimentally observed run-time statistics, event duration, and turn-angle distributions. For simulation details, we refer to the supplemental information.

**Figure 5:**
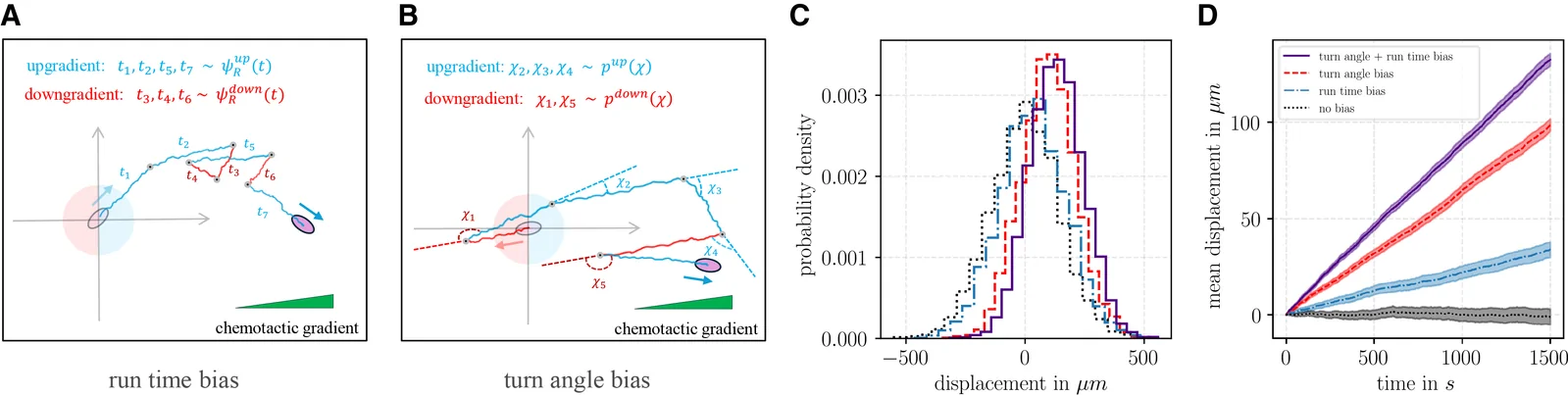
Chemotactic drift of simulated bacteria. (A and B) Schematics of agent-based simulations. Red and blue semi-circles around the origin correspond to directions of upgradient and downgradient runs, respectively. Gray dots correspond to turn and trap events. In the case of a run-time bias (A), parameters corresponding to the distributions $\psi_{R}^{up}\left(t\right)$ and $\psi_{R}^{\mathrm{down}}\left(t\right)$ are inferred directly from the experiments. In case of a turn-angle bias (B), angles corresponding to the distributions $p^{up}\left(\chi \right)$ and $p^{\mathrm{down}}\left(\chi \right)$ are sampled directly from the corresponding experimental, empirical distributions. Simulations were performed for bacteria without any chemotaxis strategy (black), with run-time bias only (blue), with turn-angle bias only (red), and with both run-time and turn-angle bias (purple). (C) Distributions of the total displacements of bacteria over the time of simulation (25 min). (D) Mean displacements as a function of time, including a 2*σ* standard error.

To include run-time and turn-angle bias in the simulations, run times and angular changes during turn events were drawn from experimentally determined distributions depending on the directions of the preceding runs with respect to the chemical gradient; results are displayed in Figure 5. We found that the average chemotactic drift of bacteria that rely solely on a turn-angle bias for their chemotactic navigation reaches approximately 72% of the drift of bacteria relying on both strategies. Bacteria that rely on a run-time bias only show a significantly slower chemotactic drift, reaching 25% of the drift of bacteria with both strategies. We thus conclude that the angle bias is the relevant mechanism governing the chemotactic performance of bacteria in porous media such as agar gels. Note that these simulations only provide conclusions regarding the efficiency of a motility bias for chemotaxis and not about the colony growth in a swimming agar assay, as the proliferation of bacteria is not included in the simulations.^17^^,^^34^

To compare the chemotactic efficiency in agar with the navigation strategy in bulk liquid, we also performed agent-based simulations, using the run-time and turn-angle statistics previously obtained from chemotaxis experiments in bulk liquid.^33^ We found that, although the turn-angle bias remains the predominant mechanism, the chemotactic drift speed of bacteria with turn-angle bias only reaches around 59% of the speed of bacteria with both strategies; in contrast, the drift speed of bacteria with run-time bias only reaches approximately 41% compared to the drift of simulated bacteria employing both strategies in bulk liquid (see supplemental information). Hence, the comparison reveals that turn-angle bias is particularly important for the overall chemotaxis performance of *P. putida* in disordered environments, in which the run length is limited by the surrounding matrix.

## Discussion

We have shown from the dynamics of a spreading culture, as well as on the level of single-cell trajectories, that *P. putida* performs chemotaxis in a porous agar gel when exposed to gradients in casamino acid concentration. We found that chemotactic navigation relies on both run-time and turn-angle biases, with the latter being the dominant contribution to the overall chemotactic drift.

### The role of turn-angle bias in bacterial chemotaxis

The turn-angle bias, as an additional chemotaxis strategy in addition to the run-time bias, was previously observed for the peritrichously flagellated bacteria *E. coli*^23^^,^^24^^,^^25^^,^^26^^,^^41^ and *Salmonella* Typhimurium^27^ during chemotaxis in aqueous bulk fluid. Also for *E. coli* chemotaxis in porous media, it was shown that runs are preferentially oriented in the gradient direction.^9^ Here, we provide the first direct experimental evidence that for *P. putida* chemotaxis in porous agar gels the change in swimming direction upon a turn event is biased depending on the orientation of the previous run with respect to the gradient (Figure 3). We demonstrate that the angular bias is induced by active, motor-triggered turn events, while reorientation events due to passive mechanical trapping in the surrounding matrix do not contribute to the turn-angle bias. Furthermore, our numerical simulations indicate that, in this crowded porous environment, the angle bias appears as the predominant chemotaxis strategy accounting for 72% of the chemotactic performance.

The small numbers we observed for the directional bias (less than 10 percentage points difference between the numbers of runs pointing toward and away from the chemoattractant gradient) are in agreement with earlier work on *E. coli* chemotaxis in porous media.^9^ In addition, earlier modeling work has shown that small adjustments in the turning angle are already sufficient to significantly improve chemotactic performance.^23^ Nevertheless, these numbers are in line with the temporal sensing paradigm of bacterial chemotaxis, where cells measure temporal changes in chemoattractant concentration while moving along their swimming trajectories and adapt their tumbling rate accordingly.^42^ This results in a biased random walk with an overall chemotactic drift speed that amounts to only a few percent of their actual swimming speed.^7^^,^^43^ We thus conclude that a turn-angle bias is crucially important for enabling bacterial chemotaxis in disordered environments, where the mean free path is reduced, limiting the impact of a run-time bias. In future work, more bacterial species shall be examined to substantiate whether chemotactic motion based on a turn-angle bias is commonly observed in bacterial chemotaxis.

### Flagellar dynamics and the mechanistic basis of turn-angle bias

During run-and-tumble motility of *E. coli*, run phases of persistent forward motion are observed when all flagella rotate counterclockwise. Runs are interrupted by tumble events, during which one or several flagella switch to clockwise rotation. By this, the flagella bundle falls apart, and bacteria change their body orientation. The angular change during a tumble depends on the number of filaments that leave the flagellar bundle^44^: the more flagella switch to clockwise rotation during a tumble, the larger, on average, the angular change. To induce chemotactic drift by tuning the tumble angle, the probability that a flagellum switches to clockwise rotation has to depend on the run direction with respect to the chemoattractant gradient. If *E. coli* swims downgradient, the probability of switching to clockwise rotation increases, thereby increasing the turning angle. When moving upgradient, fewer flagella switch to clockwise rotation, so the angular change during a tumble will be smaller. In this scenario, run-time bias and turn-angle bias are closely related because a higher probability of switching to clockwise rotation also leads to shorter runs in the downgradient direction.^23^

Here, we have provided direct evidence for the first time, to our knowledge, that the chemotaxis strategy of the lophotrichously flagellated bacterial swimmer *P. putida* is predominantly determined by turn-angle bias. Thus, actively biasing the turning angle is not a unique feature of peritrichously flagellated bacteria but also plays a key role during chemotaxis of polarly flagellated species. However, compared to *E. coli*, the motility pattern of *P. putida* is more complex; therefore, the mechanism of turn-angle bias proposed for *E. coli* cannot be directly transferred to *P. putida*. *P. putida* swims in runs interrupted by stops and reversals. During run phases, the flagellar bundle may rotate counterclockwise to push the cell body forward, it may rotate clockwise to pull the cell body, or it may wrap around it to induce a screw thread motion of the cell.^32^^,^^45^ To induce transitions between these swimming modes, the flagellar motors have to switch between counterclockwise (push) and clockwise (pull/wrapped) rotation, or they have to change from low (pull) to high (wrapped mode) motor torque.^32^^,^^46^ We hypothesize that the turning angle during these transitions may depend on the degree of synchrony at which the motors are changing their torque or sense of rotation; in consequence, the statistics of transitions between swim modes (push, pull, and wrapped) may change in such a way that the ratio of stop and reversal events effectively depends on the direction of motion with respect to the chemoattractant gradient. This is supported by recent numerical observations showing that a mismatch in motor torques may induce a rich variety of exotic swimming modes, some of which have been occasionally seen in experiments.^47^ Moreover, stop events may also be triggered by asynchronous motor activity. In particular, for stops that interrupt run episodes in wrapped mode, a broad turn-angle distribution was observed.^33^

In the standard model of chemotaxis, bacteria are assumed to measure a chemical gradient by comparing nutrient concentrations along their swimming path, a process referred to as temporal sensing.^48^^,^^49^^,^^50^^,^^51^^,^^52^^,^^53^ An open question arises when considering porous media in which swimming phases are short, as it is the case for the media examined here: the mean run duration is approximately 0.2 s in 0.3% agar (Table 1), and runs are interrupted by traps, which are at least one order of magnitude longer on average.^14^ Our findings indicate, however, that bacteria do nevertheless perform chemotaxis in these porous environments. In the future, it will be important to ascertain in detail whether these short swimming intervals, accompanied by long-lasting traps, are compatible with the current understanding of bacterial chemotaxis based on temporal sensing. It will also be interesting to see how other species of bacteria with different flagellation patterns perform chemotaxis in porous media, compared with *E. coli* and *P. putida*. As a first step in this direction, we also recorded the trajectories of two *P. putida* knockout mutants, deficient in the MotAB or MotCD stators of the flagellar motor.^13^^,^^14^ Both mutant cell lines were recorded in 0.25% agar, and the same analysis toolbox was applied to compare their chemotactic efficiencies with wild-type cells as described previously. The analysis revealed that the overall chemotactic performances of the two mutants, as quantified by drift velocity in agent-based simulations, were similar to those of wild-type cells. However, the relative contribution of turn-angle bias in comparison to run-time bias for the mutant cells increases significantly. For details of our analysis and the corresponding figures, see supplemental information.

Taken together, our results demonstrate that the lophotrichously flagellated soil bacterium *P. putida* primarily relies on a turn-angle bias to navigate along chemical gradients in porous gel-like matrices. We hypothesize that the degree of synchrony in motor operation during turning maneuvers provides the mechanistic basis for active turn-angle regulation in the chemotaxis of this polarly flagellated swimmer.

## Data availability

The raw data that support the findings of this study and the computer codes used for data analysis and numerical simulations are available from the corresponding author upon reasonable request. The analyzed trajectories of *P. putida* in agar are available at https://doi.org/10.5281/zenodo.17592316.

## Acknowledgments

We acknowledge Kolja Klett, Hans Reimann, and Jan Albrecht for helpful discussions. This research was partially funded by 10.13039/501100001659Deutsche Forschungsgemeinschaft (DFG), project IDs 443369470-BE 3978/13-1 (V.P. and C.B.) and 318763901 – SFB1294 (A.D., S.B., R.G., and C.B.).

## Author contributions

S.B. and V.P. conducted the experiments. S.B. and A.D. processed the experimental data and performed the analysis. A.D. performed the simulation. S.B., V.P., A.D., and C.B. wrote the manuscript. R.G. and C.B. supervised the project. All authors discussed the results and contributed to the final manuscript.

## Declaration of interests

The authors declare no competing interests.

## Supplemental information

Document S1. Figures S1–S4, Tables S1–S5, and supplemental materials and methods

Document S2. Article plus supplemental information
