# Supplementary material for: Dual chemotaxis strategy of bacteria in porous media: Run-time and turn-angle bias
Source: Biophys J. 2026 Jun 4;125(14):3570–9. doi: 10.1016/j.bpj.2026.06.002 (PMC13507469; doi:10.1016/j.bpj.2026.06.002)
Supplement: Supplementary file 1 [file mmc1.pdf]

Biophysical Journal, Volume *125*

Supplemental information

Dual chemotaxis strategy of bacteria in porous media: Run-time and turn-angle bias

Sönke Beier, Veronika Pfeifer, Agniva Datta, Robert Großmann, and Carsten Beta

SUPPLEMENTARY MATERIAL

Dual Chemotaxis Strategy of Bacteria in Porous Media: Run Duration and Turning Angle Bias

Sönke Beier, Veronika Pfeifer, Agniva Datta, Robert Großmann and Carsten Beta

AGENT BASED SIMULATIONS: CHEMOTAXIS IN DISORDERED ENVIRONMENTS

Here details of numerical simulations of bacterial motility in disordered environments, with a particular focus on the relevance of run time and turn angle bias for the overall chemotactic performance are summarized. All simulations are based on the modeling approach discussed in Refs. [1, 2].

Based on experimental observations, we describe the motility of the bacteria to be comprised of two phases, namely a self-propelled run phase and a turn phase representing the events (both, mechanical traps and actively triggered reorientations) during which self-propulsion is absent. Mathematically, we describe this motility by the following set of coupled Langevin equations:

$$\dot{\mathbf{r}}(t) = v(t)\begin{pmatrix}\cos\phi(t)\\ \sin\phi(t)\end{pmatrix}, \tag{S1a}$$

$$\dot{\phi}(t) = \sqrt{2D_\phi(t)}\,\xi(t) + \zeta_\chi(t). \tag{S1b}$$

The velocity $v(t)$ and the rotational diffusion coefficient $D_\phi(t)$ are given by (v_0, D_ϕ) in the run phase and $(0,0)$ in the turn phase as the spatial displacements and angular fluctuations during events are negligible compared to those in the run phase. The second term in Eq. (S1b), i.e. $\zeta_\chi(t)$, corresponds to a non-Poissonian shot noise: at the end of each turn phase, the bacterium randomly reorients with respect to its last orientation of the body axis,

$$\phi \to \phi + \chi, \tag{S2}$$

in which the angle χ is drawn from a turn angle distribution $p(\chi)$.

The durations of run and turn phases are drawn from the experimentally observed waiting time distributions which we infer by nonparametric likelihood estimation [3] and subsequently fit with a parametric form, similar to Ref. [2]. For the fit, we parametrize the run time distribution $\psi_R(t)$ by a double exponential distribution with a minimal run time,

$$\psi_R(t) = \begin{cases} 0 & t \leq t_0, \\ p\kappa e^{-\kappa(t-t_0)} + (1-p)\lambda e^{-\lambda(t-t_0)} & t > t_0, \end{cases} \tag{S3}$$

and the dwell time distributions $\psi_T(t)$ by a piecewise power law

$$\psi_T(t) = \begin{cases} 0 & t < \tau_1, \\ At^{-\alpha} & t \in (\tau_1, \tau_2), \\ Bt^{-\beta} & t > \tau_2, \end{cases} \quad A = \frac{(\alpha-1)(\beta-1)(\tau_1\tau_2)^\alpha}{(\beta-1)\tau_1\tau_2^\alpha + (\alpha-\beta)\tau_1^\alpha\tau_2}, \quad B = A{\tau_2}^{\beta-\alpha} \tag{S4}$$

in which the parameters A and B ensure the continuity at $t = \tau_2$ and normalization of $\psi_T(t)$. We inferred the following parameters of the dwell time distribution $\psi_T(t)$: $\tau_1 = 0.03\,\mathrm{s}$, $\tau_2 = 2.95\,\mathrm{s}$, $\alpha = 0.82$, $\beta = 2.64$. Considering the motility data in $0.25\,\%$ agar, we further estimated the run velocity $v_0 = 19.49\,\mu\mathrm{m/s}$ as well as the rotational diffusion coefficient $D_\phi = 0.29\,\mathrm{s}^{-1}$ which we obtain from the experimental data by the methods described in Ref. [2]. All simulations are initialized in the run state with an initial orientation angle sampled from an uniform distribution in the interval $[-\pi, \pi)$ so that the motion is equally likely to start up- and downgradient.

We simulate 5000 trajectories (total duration: 25 min) with a time step of $\Delta t = 0.05\,\mathrm{s}$, considering four different chemotaxis strategies as described in the main text: (i) no bias; (ii) run time bias only; (iii) angle bias only; (iv) run time and angle bias. Below, we describe the four different chemotaxis strategies separately.

(i) No bias. If neither run durations nor turn angles depend on the direction of motion, there is no chemotactic drift. We use this case as a control. In this setting, the run time distribution $\psi_R(t)$ is estimated from all observed run episodes. The following parameters were obtained: $t_0 = 0.08\,\mathrm{s}$, $\kappa = 3.94\,\mathrm{s}^{-1}$, $\lambda = 7.23\,\mathrm{s}^{-1}$, $p = 0.49$. Run times are sampled from the distribution ψ_R, Eq. (S3), by inverse transform sampling. Turn angles are directly sampled uniformly from the list of experimentally observed values.

(ii) Run time bias only. Here, we distinguish the runs into runs pointing upgradient and those oriented downgradient, and fit the probability distribution functions separately to obtain the parameter values summarized in Tab. S1. Turn angles are sampled from the list of experimentally observed values, analogous to (i).

	t_0	κ	λ	p
all runs	$0.08\,\mathrm{s}$	$3.94\,\mathrm{s}^{-1}$	$7.23\,\mathrm{s}^{-1}$	0.49
upgradient runs	$0.08\,\mathrm{s}$	$7.46\,\mathrm{s}^{-1}$	$3.85\,\mathrm{s}^{-1}$	0.52
downgradient runs	$0.08\,\mathrm{s}$	$3.81\,\mathrm{s}^{-1}$	$6.57\,\mathrm{s}^{-1}$	0.29

Table S1. Fit parameters of the run time distribution ψ_R for upgradient and downgradient runs.

(iii) Turn angle bias only. To simulate bacteria with turn angle bias only, we use the run time distribution as described in (i). Turn angles are first distinguished by the turn durations t_T into short ($t_T < 1\,\mathrm{s}$) and long events ($t_T > 1\,\mathrm{s}$), as described in the main text (Fig. 3). Moreover, we further distinguish them into two subgroups based on the orientation of the preceding run (upgradient or downgradient). The actual values for the simulation are sampled from the list of experimentally recorded data. In short, we sample turn angles from four different types of events: short events following an (a) upgradient or (b) downgradient run and long events following an (c) upgradient or (d) downgradient run.

(iv) Run time and turn angle bias. Here, we combine both strategies and, therefore, use two run time distributions similar to strategy (ii). Moreover, we sort turn angles into four categories as described in (iii).

AGENT BASED SIMULATIONS: RUN-AND-TURN MOTILITY IN BULK

In order to assess the relevance of run time and turn angle bias of bacteria in bulk liquid, we simulate trajectories based on a previously recorded experimental dataset: *P. putida* in bulk fluid under the influence of an external, linear chemoattractant gradient [4]. For bacteria swimming in liquid, the duration of turn phases is negligible—run times are typically one order of magnitude longer than turns [4]. The model then simplifies to the classical run-and-tumble motility with approximately exponentially distributed run times [4], modified here by a minimal run time t_0:

$$\psi_R(t) = \begin{cases} 0 & t \le t_0, \\ \lambda e^{-\lambda(t-t_0)} & t > t_0. \end{cases} \tag{S5}$$

Run time distributions were obtained by nonparametric maximum likelihood estimation and subsequent fitting as described in the previous section. The fit parameters are summarized in Tab. S2. The minimal run time t_0 in Eq. (S5) was introduced to better fit the inferred distributions; it is typically of the order of the time interval between two consecutive frames ($0.1\,\mathrm{s}$).

	t_0	λ
all runs; no gradient	$0.04\,\mathrm{s}$	$0.92\,\mathrm{s}^{-1}$
upgradient runs	$0.15\,\mathrm{s}$	$0.99\,\mathrm{s}^{-1}$
downgradient runs	$0.15\,\mathrm{s}$	$0.88\,\mathrm{s}^{-1}$

Table S2. Fit parameters for run time distributions $\psi_R(t)$ in bulk for upgradient runs, downgradient runs and runs in a homogeneous environment (without a gradient).

Further, reorientation angles (tumbles) are directly sampled from the experimentally observed empirical distributions. Note that long events are absent for bacterial swimmers in bulk liquid. The velocity and rotational diffusion coefficient are estimated to be $v_0 = 18.75\,\mu\mathrm{m/s}$ and $D_\phi = 0.35\,\mathrm{s}^{-1}$ following the methodology outlined in Ref. [2]. We simulated 5000 trajectories (total length: $300\,\mathrm{s}$) with a time step of $\Delta t = 0.1\,\mathrm{s}$. Fig. S1 summarizes the main result: we plot the distribution of total displacements within the total simulation time along with the mean displacement of the tracks as a function of time for four different settings: (i) no bias; (ii) run time bias only; (iii) angle bias only; (iv) run time and angle bias.

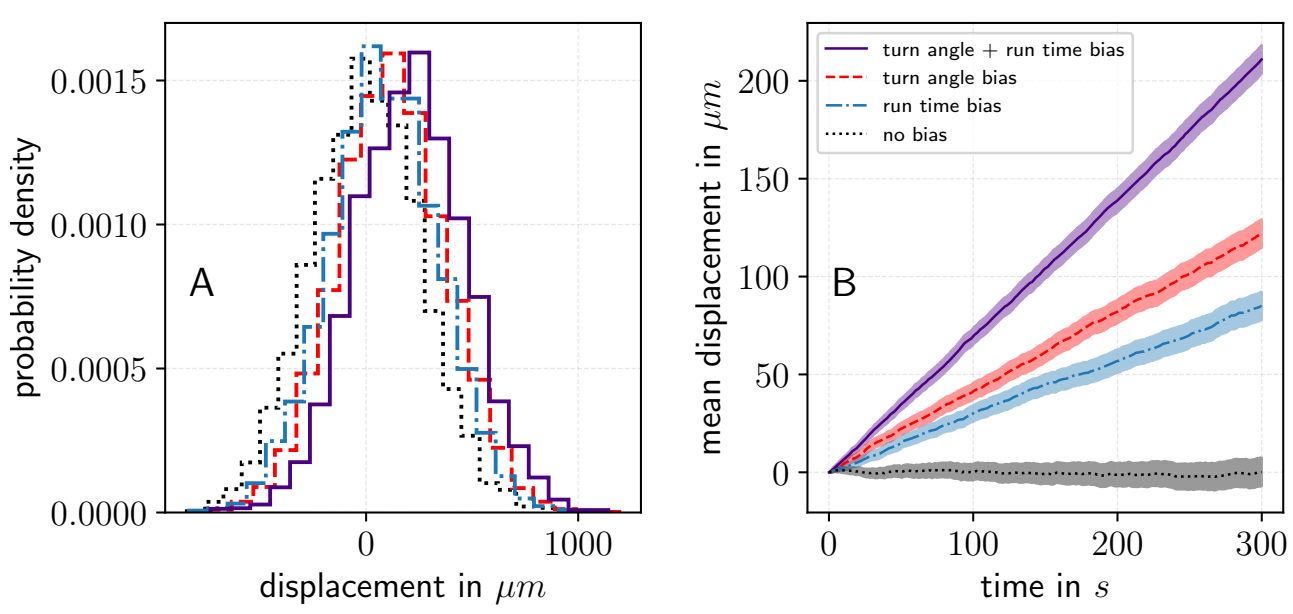

Figure S1. **Chemotactic drift of simulated bacteria in bulk liquid.** Simulations were performed for bacteria without any chemotaxis strategy (black), with run time bias only (blue), with turn angle bias only (red) and with both, run time and turn angle bias (purple). Panel A: distribution of the total displacements of bacteria within a time window of $300\,\mathrm{s}$. Panel B: mean displacement of bacteria as a function of time, with a 2σ standard error.

CHEMOTACTIC BEHAVIOR OF STATOR KNOCKOUT MUTANTS

In addition to the wild type cells of *P. putida*, we here analyze the chemotactic behavior of mutants deficient in the MotAB or MotCD stators of the flagellar motor, respectively. The motility of these mutant cell lines was characterized in Ref. [5], where it was observed that the distribution of run types (push, pull, and wrapped) is altered in these mutants. For Δ*motAB* cells, the pull mode is more frequent, while the wrapped mode occurs less often. The distribution of run modes for ΔmotCD cells is similar to that of the wild type. Additionally, it is known from Ref. [4] that in a chemoattractant gradient, the push mode does not exhibit a runtime bias, whereas the wrapped mode shows a runtime bias. The pull mode occurred only rarely and therefore could not be analyzed.

In figure S2 the run time bias (A), the run orientation preference (B, C), and the angle bias depending on the orientation of the preceding run (D, E, F, G) show that both mutants are performing chemotaxis. A weak runtime bias can be seen for both mutants. In contrast, the angular bias differs between the mutants, with Δ*motAB* cells displaying a stronger angular bias than Δ*motCD* cells (compare figures S2E and G).

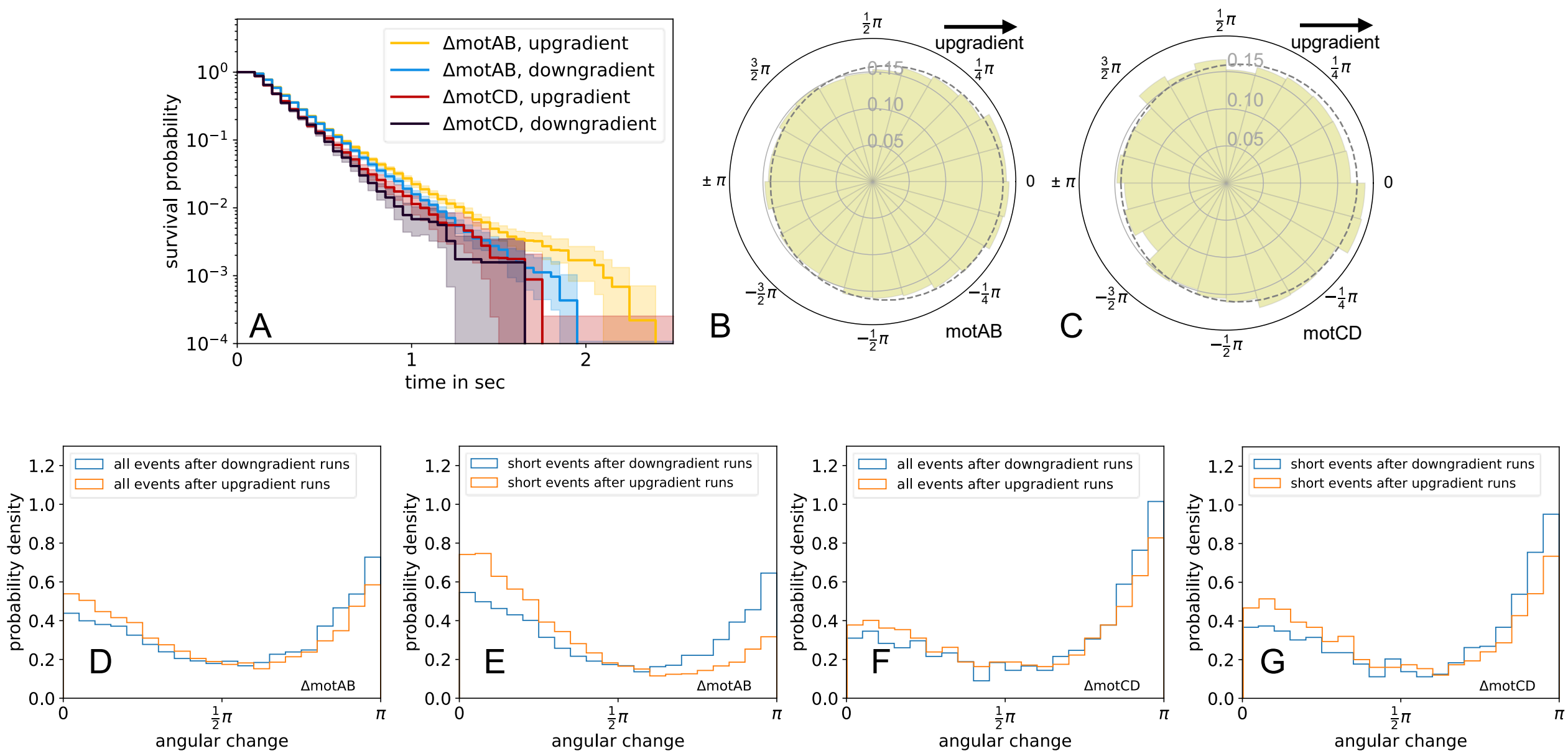

Figure S2. **Chemotaxis of motAB and motCD stator knockout mutants.** For ΔmotAB 19893 trajectories were analyzed. For ΔmotCD 4020 trajectories were analyzed **A**: Run time distributions show a small run time bias for both mutants. **B and C:** The distributions of run orientations show orientational preference of runs for both mutants. For ΔmotAB cells $53.8 \pm 0.3\%$ of runs are orientated upgradient. For ΔmotCD cells $53.2 \pm 0.6\%$ of runs are oriented upgradient. **D,E,F and G:** The distributions of turn angles during event phases are showing a turn angle bias for both mutants. Restricting the analysis to the actively triggered short events yields a larger effect because the effect of trap events caused by the agar network is substracted.

We also performed agent-based simulations by fitting the run-time and dwell-time distributions, and inferring the velocity and rotational diffusion coefficient (see tables S3, S4, S5), and obtaining the turn angles sampled uniformly from the list of experimentally observed values following the same method as used for the WT cells presented in the main text. We find that the simulation results are consistent with our experimentally observed statistics as shown in figure S2.

The simulations revealed that although the overall chemotactic performances (incorporating both run-time and turn angle bias) for the two mutants are similar to the WT, the relative contribution of the turn-angle bias increases significantly in both cases (see figures S3, S4). For Δ*motAB* and Δ*motCD*, we find that the relative percentages of the drift velocity reached by only the turn-angle bias, in comparison to the drift velocity reached by both strategies, are around 88% and 85%, respectively, compared to 72% as observed in the WT cells (see main text).

	t_0	κ	λ	p
all runs	0.10 s	3.12 s^{-1}	6.21 s^{-1}	0.31
upgradient runs	0.09 s	2.27 s^{-1}	5.23 s^{-1}	0.11
downgradient runs	0.09 s	2.81 s^{-1}	5.10 s^{-1}	0.08

Table S3. Fit parameters of the run time distribution ψ_R corresponding to Δ*motAB* mutant for upgradient and downgradient runs.

	t_0	κ	λ	p
all runs	0.07 s	3.47 s^{-1}	6.42 s^{-1}	0.23
upgradient runs	0.07 s	7.35 s^{-1}	3.76 s^{-1}	0.64
downgradient runs	0.08 s	4.01 s^{-1}	6.59 s^{-1}	0.24

Table S4. Fit parameters of the run time distribution ψ_R corresponding to Δ*motCD* mutant for upgradient and downgradient runs.

**strain**	v_0	D_ϕ	ψ_T [Eq. (S4)]			
			τ_1	τ_2	α	β
$\Delta motAB$	16.64	0.51	0.03	4.01	0.83	3.19
$\Delta motCD$	23.46	0.33	0.03	2.56	0.75	2.58

Table S5. The table summarizes all inferred parameter values, obtained from the analysis of experimental data as described in Ref. [2] for the two mutants cells Δ*motAB* and Δ*motCD* in $0.25\,\%$ agar. Parameters are expressed in the following units: v_0 in µm/s; D_ϕ in s^{-1}; τ_1 and τ_2 in s.

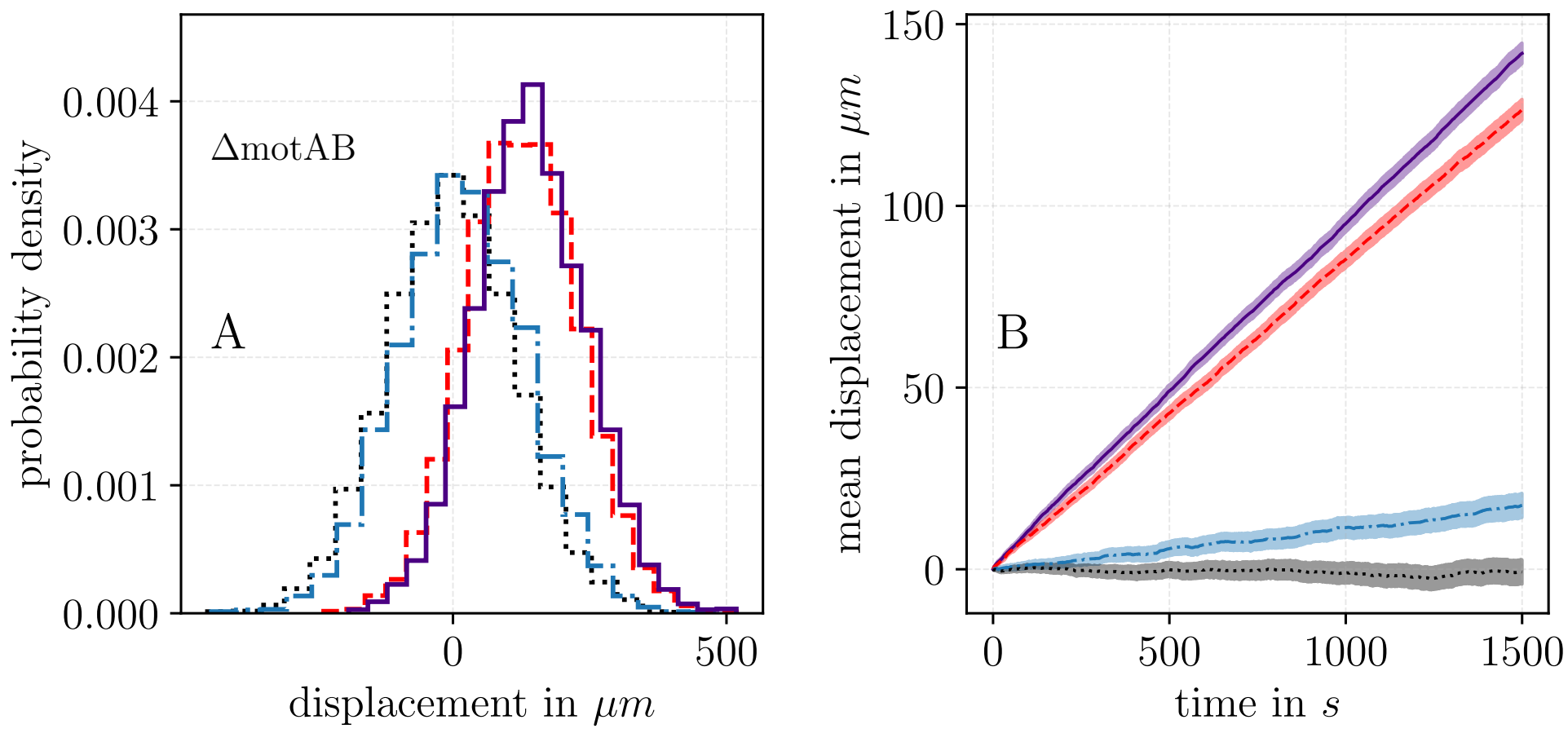

Figure S3. **Simulations of chemotactic drift corresponding to mutant Δ *motAB*** Simulations were performed for bacteria without any chemotaxis strategy (black), with run time bias only (blue), with turn angle bias only (red) and with both, run time and turn angle bias (purple). Panel A: distribution of the total displacements of bacteria within a time window of 1500 s. Panel B: mean displacement of bacteria as a function of time, with a 2σ standard error.

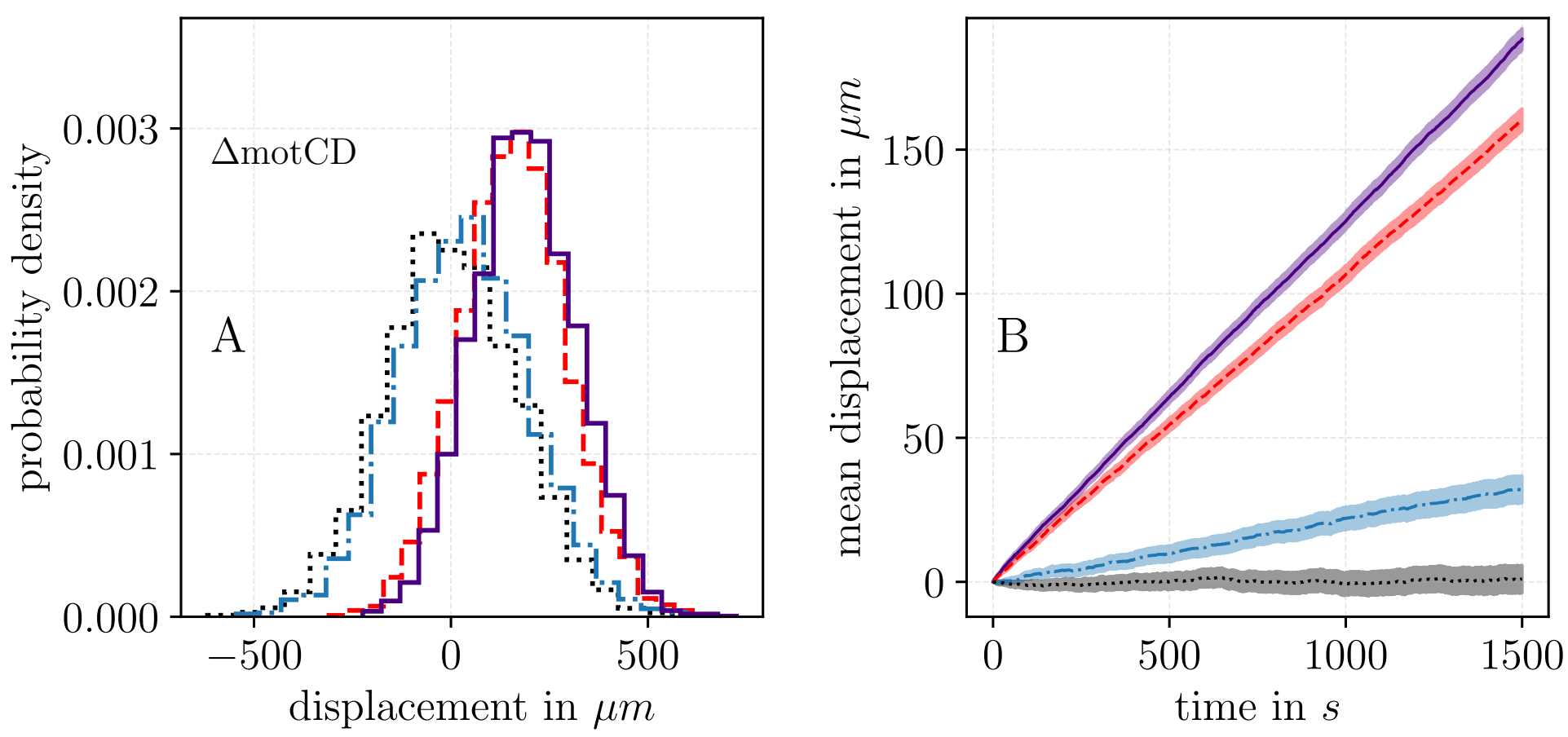

Figure S4. **Simulations of chemotactic drift corresponding to mutant Δ *motCD*** Simulations were performed for bacteria without any chemotaxis strategy (black), with run time bias only (blue), with turn angle bias only (red) and with both, run time and turn angle bias (purple). Panel A: distribution of the total displacements of bacteria within a time window of 1500 s. Panel B: mean displacement of bacteria as a function of time, with a 2σ standard error.

[1] A. Datta, C. Beta, and R. Großmann, Random walks of intermittently self-propelled particles, Phys. Rev. Res. **6**, 043281 (2024).

[2] A. Datta, S. Beier, V. Pfeifer, R. Großmann, and C. Beta, Bacterial swimming in porous gels exhibits intermittent run motility with active turns and mechanical trapping, Sci. Rep. **15**, 20320 (2025).

[3] Y. Vardi, Nonparametric estimation in renewal processes, Ann. Stat. **10**, 772 (1982).

[4] Z. Alirezaeizanjani, R. Großmann, V. Pfeifer, M. Hintsche, and C. Beta, Chemotaxis strategies of bacteria with multiple run modes, Sci. Adv. **6**, eaaz6153 (2020).

[5] V. Pfeifer, S. Beier, Z. Alirezaeizanjani, and C. Beta, Role of the two flagellar stators in swimming motility of *Pseudomonas putida*, mBio **13**, e02182-22 (2022).
